# Comorbid infections induce progression of visceral leishmaniasis

**DOI:** 10.1186/s13071-019-3312-3

**Published:** 2019-01-23

**Authors:** Angela J. Toepp, Glória R. G. Monteiro, José F. V. Coutinho, Adam Leal Lima, Mandy Larson, Geneva Wilson, Tara Grinnage-Pulley, Carolyne Bennett, Kurayi Mahachi, Bryan Anderson, Marie V. Ozanne, Michael Anderson, Hailie Fowler, Molly Parrish, Kelsey Willardson, Jill Saucier, Phyllis Tyrell, Zachary Palmer, Jesse Buch, Ramaswamy Chandrashekar, Grant D. Brown, Jacob J. Oleson, Selma M. B. Jeronimo, Christine A. Petersen

**Affiliations:** 1 Department of Epidemiology, College of Public Health, University of Iowa, Iowa City, Iowa 52242 USA; 2 Center for Emerging Infectious Diseases, University of Iowa Research Park, Coralville, Iowa 52241 USA; 3 Institute of Tropical Medicine of Rio Grande do Norte, Federal University of Rio Grande do Norte, Natal, RN 59078-970 Brazil; 4 Department of Biostatistics, College of Public Health, University of Iowa, Iowa City, Iowa 52242 USA; 5 Immunology Program, Carver College of Medicine, University of Iowa, Iowa City, Iowa 52242 USA; 6 IDEXX Laboratories Inc., One IDEXX Drive, Westbrook, Maine 04092 USA; 7 Department of Geographical and Sustainability Sciences, College of Liberal Arts & Sciences, University of Iowa, Iowa City, Iowa 52242 USA

**Keywords:** Tick-borne diseases, Canine leishmaniosis, Risk-factor, Progression

## Abstract

**Background:**

Visceral leishmaniasis (VL) is a vector borne zoonotic disease endemic in humans and dogs in Brazil. Due to the increased risk of human infection secondary to the presence of infected dogs, public health measures in Brazil mandate testing and culling of infected dogs. Despite this important relationship between human and canine infection, little is known about what makes the dog reservoir progress to clinical illness, significantly tied to infectiousness to sand flies. Dogs in endemic areas of Brazil are exposed to many tick-borne pathogens, which are likely to alter the immune environment and thus control of *L. infantum*.

**Results:**

A cross-sectional study of 223 dogs from an area of Natal, in the Rio Grande do Norte, Brazil, were studied to determine the association between comorbid tick-borne disease and *Leishmania* infection in this endemic area. The risk of *Leishmania* seropositivity was 1.68× greater in dogs with tick-borne disease seropositivity compared to those without (Adjusted RR: 1.68, 95% CI: 1.09–2.61, *P* = 0.019). A longitudinal study of 214 hunting dogs in the USA was conducted to determine the causal relationship between infection with tick-borne diseases and progression of VL. Hunting dogs were evaluated three times across a full tick season to detect incident infection with tick-borne diseases. A logistic regression model with generalized estimating equations to estimate the parameters was used to determine how exposure to tick-borne disease altered VL progression over these three time points when controlling for other variables. Dogs infected with three or more tick-borne diseases were 11× more likely to be associated with progression to clinical VL than dogs with no tick-borne disease (Adjusted RR: 11.64, 95% CI: 1.22–110.99, *P* = 0.03). Dogs with exposure to both *Leishmania* spp. and tick-borne diseases were five times more likely to die during the study period (RR: 4.85, 95% CI: 1.65–14.24, *P* = 0.0051).

**Conclusions:**

Comorbid tick-borne diseases dramatically increased the likelihood that a dog had clinical *L. infantum* infection, making them more likely to transmit infection to sand flies and people. As an important consequence, reduction of tick-borne disease exposure through topical or oral insecticides may be an important way to reduce progression and transmissibility of *Leishmania* infection from the canine reservoir to people.

**Electronic supplementary material:**

The online version of this article (10.1186/s13071-019-3312-3) contains supplementary material, which is available to authorized users.

## Background

Vector borne zoonotic diseases remain important public health concerns across the globe. No infection occurs in isolation, instead comorbid infection(s) can alter the inflammatory state and subsequently disease outcome [1]. *Leishmania* spp. are obligate intracellular protozoan parasites which cause a spectrum of diseases, ranging from focal cutaneous lesions to visceralizing disease, endemic within 98 countries across the globe [2]. Visceral leishmaniasis (VL), caused by zoonotic *L. infantum*, affects nearly 300,000 people each year and leads to more than 30,000 deaths annually. In South America, *L. infantum* is transmitted both vertically and *via* phlebotomine sand flies, most commonly *Lutzomyia longipalpis* [3–5]. In Brazil alone there are more than 3000 new human cases of visceral leishmaniasis each year, with many more likely cases unreported [6]. Disease prevalence in the residents of Natal directly echoes seropositivity of dogs in the area [7], highlighting that disease is often maintained in the canine reservoir and is spread *via* sand flies to nearby people.

VL is enzootic in Brazil’s dog population, the domestic reservoir of *L. infantum*. Similar to other locations with zoonotic VL [8–10], infected dog ownership remains a significant risk factor predisposing humans to infection [11]. Seroprevalence of canine leishmaniasis (CanL) in Natal has been reported to be between 20–33% [7, 12]. Because of the important role of dogs as a domestic reservoir for *L. infantum* in Brazil, current federal public health policy dictates that household dogs are tested for *L. infantum via* a serological snap test and if positive are required to be donated for euthanasia by the Center of Zoonotic Diseases for each region [13]. Both dogs and humans have a wide range of clinical presentation due to infection with *L. infantum*, ranging from asymptomatic to fatal visceralizing disease. Host and parasite factors that determine clinical outcome are poorly understood. More attention and a better understanding of this neglected disease, particularly the factors that predict clinical outcome, is needed to better control and prevent spread of *L. infantum*.

Within the USA, leishmaniosis is enzootic within the hunting dog population with a qPCR prevalence of 20%, similar to rates of seroprevalence seen in countries with established sand fly-transmitted disease [14, 15]. CanL in the USA is transmitted vertically with no documented vector transmission [4, 16]. This USA cohort is unique as dogs are exposed to *Leishmania* parasites *in utero* then exposed to tick-borne co-infections throughout their lifetime, allowing temporal examination and causal determination of the effects of tick-borne co-infection upon the progression of already present *L. infantum* infection. Tick-borne diseases are prevalent in people and animals across the USA. Prevalence of tick-borne co-infections in this canine research cohort is much greater than that of the USA dog population [17]. For instance, the average seroprevalence of *Anaplasma* spp. is estimated to be 4.8% overall in USA dogs [17] while this hunting dog population had a prevalence almost five times higher, near 25% (Mahachi et al., unpublished data).

The overlapping immune cellular tropisms of tick-borne pathogens and *L. infantum* with alteration to the immune response needed to control of *L. infantum* infection suggests that there is biological plausibility that tick-borne co-infections would alter the immune balance during subclinical VL prompting progression to clinical leishmaniosis.

Co-infection of tick-borne pathogens, common exposures to dogs at high risk of CanL, has been previously reported [18, 19]. Due to a lack of longitudinal data, previous studies could not assess the causal relationship of comorbid infection to risk of CanL progression [20]. Here we assessed how exposure to tick-borne co-infection altered the clinical outcome of *L. infantum* infection *via* first a cross-sectional study performed in an endemic area of Brazil. Based on these findings, to solidify the correlation between tick-borne infection and timing of progression to clinical CanL, we obtained temporal data for tick-borne disease exposure and CanL in a longitudinal case-control study. The hypothesis was that dogs exposed to numerous tick-borne co-infections have a higher relative risk of progression to VL.

## Methods

### Study design: Brazil

Two hundred twenty-three dogs were enrolled in a cross-sectional study of household dogs to determine the seroprevalence of tick-borne co-infections with *Leishmania* spp. in peri-urban areas of Natal, Rio Grande do Norte, Brazil [21, 22]. All dogs were visually assessed by veterinarians and tested serologically for exposure to *Anaplasma* spp., *Ehrlichia* spp. and *Leishmania* spp. All dogs were included in the study with no restrictions. All dogs in the study had similar outdoor exposures and no active intervention for CanL. This is an area, like much of Brazil, where active surveillance is performed to diagnose CanL and the Centro de Controle de Zoonoses (CDZ) euthanizes dogs found to be seropositive for CanL on two tests in accordance with Ministry of Health guidelines.

### Study design: USA

Two hundred eleven dogs, a subset from a block-randomized, double-blinded, placebo-controlled, vaccine trial, were followed longitudinally over a ten-month period (Additional file 1: Figure S1) [23]. Dogs were tested for leishmaniosis *via* qPCR, DPP® CVL assay [24], and a physical exam. Dogs were tested *via* qPCR and serology for tick-borne disease co-infections. Dogs were included in the study if they were older than 6 months of age, not pregnant and not clinically ill with leishmaniasis or any tick-borne disease at the time of study enrollment. All enrollment criteria are the same as our field vaccine trial described in Toepp et al. [25]. Beyond vaccination, which was a covariate analyzed in the present studies, no additional interventions were in place to reduce CanL.

### *Leishmania* status: Brazil

Exposure to *Leishmania* spp. was determined through three different serological tests. The Dual Path Platform® Canine Visceral Leishmaniasis (DPP® CVL) assay was used to detect exposure to *Leishmania* spp. [26]. Two enzyme-linked immunosorbent assays (ELISA) were performed to also identify *Leishmania* exposure. These two assays targeted recombinant k39 or soluble *Leishmania* antigens (SLA). Dogs were initially identified as *L. infantum*-seropositive for univariate analyses if they tested positive on any of these serological assays (DPP®CVL assay, SLA ELISA or k39 ELISA), with additional analyses using the diagnostic step-wise procedure of DPP with confirmative serology to indicate *L. infantum*-positivity [26].

### *Leishmania* status: USA

Quantitative polymerase chain reaction and the Dual Path Platform® Canine Visceral Leishmaniasis (DPP® CVL) assay were utilized to determine the *Leishmania* molecular and serological status of dogs in the USA. qPCR and DPP® CVL was performed as in [24].

### Physical examination: Brazil

Veterinarians assessed through visual examination whether dogs had signs of VL. This included onychogryphosis, cachexia, apathy and physical wounds. Blood and serum samples were collected in ethylenediaminetetraacetic acid tubes and stored at -80 °C. Serum was stored at -20 °C.

### Physical examination: USA

A veterinarian completed all physical exams. Caretakers provided information on recent travel and hunting activities, use for breeding, and any notable change in the dog’s overall health since last visit. If a dog died between visits, a caretaker provided information regarding history of observed clinical signs prior to death. When possible, a full necropsy was performed by a member of the veterinary study team to confirm cause of death with tissue samples submitted for confirmatory *Leishmania* qPCR. Clinical signs of leishmaniasis included: lymphadenopathy, spleno- and hepatomegaly, epistaxis, alopecia, characteristic macular or papular skin lesions, poor hair coat, cachexia as measured by low body condition score compared to rest of group, conjunctivitis, and onychogryphosis [27, 28].

### Outcomes: Brazil

The objective of the study in Brazil was to determine how exposure to co-infection with tick-borne diseases is associated with exposure to *Leishmania* spp. In order to assess this relationship *Leishmania* spp. exposure was the main outcome.

### Outcomes: USA

The objective of the study was to determine how exposure to co-infection with tick-borne diseases affect the progression of leishmaniosis. In order to assess clinical progression of leishmaniosis, dogs were given a clinical score; a combination of VL signs count, DPP®CVL, and qPCR for *Leishmania*. Dogs with three or more clinical signs for VL who tested positive *via* DPP®CVL and/or qPCR for *Leishmania* were identified as polysymptomatic for CanL. Dogs with two or less clinical signs positive for DPP®CVL and/or qPCR were classified as asymptomatic. Dogs negative for both DPP®CVL and qPCR were classified as negative for CanL. As leishmaniosis is an immunosuppressive disease that is fatal if untreated [29, 30] mortality was assessed during the ten-month study. All-cause mortality was defined as mortality for any reason. Licensed veterinarians established leishmaniasis-related deaths by previous *Leishmania*-specific diagnostic results, history, and clinical signs as well as immediate cause of death (kidney failure etc.).

### Measurement of tick co-infections: Brazil

Serum from dogs were tested for antibody responses to *Ehrlichia ewingii* and *Ehrlichia canis*, *Anaplasma phagocytophilum* and *Anaplasma platys*, *Borrelia burgdorferi* and *Dirofilaria immitis* antigen *via* the IDEXX SNAP® 4Dx® Plus Test. Longitudinal study and confirmation by IDEXX *via* ELISA was not possible for the Brazil arm of this work, given the need to euthanize any dog that was serologically positive at the (CDZ) in accordance with the Ministry of Health, Brazil.

### Measurement of tick co-infections: USA

Overall the method of establishing tick-borne disease exposure was the same between the two studies, IDEXX SNAP® 4Dx® Plus Test. Blood from dogs was tested *via* qPCR for the following tick-borne disease co-infections by IDEXX laboratories: *Babesia canis vogeli*, *Babesia gibsoni*, *Babesia conradae*, *Bartonella* spp., *Rickettsia* spp*.*, *Hepatozoon americanum*, *Hepatozoon canis*, *Ehrlichia canis*, *Ehrlichia chaffeensis*, *Ehrlichia ewingii*, *Anaplasma platys* and *Anaplasma phagocytophilum*. Confirmatory serological testing was performed *via* ELISA for: speciation for *Ehrlichia canis*, *Ehrlichia chaffeensis* and *Ehrlichia ewingii*; speciation for *Anaplasma platys* and *Anaplasma phagocytophilum*; speciation for *Babesia gibsonii*; *Borrelia burgdorferi*. ELISA results were used for this longitudinal study.

### USA data management

All blood and serum samples were obtained and stored with unique barcode identifiers. All dog names and matching barcode identifier numbers were securely stored on a password-protected network drive in Microsoft Excel spreadsheets and Research Electronic Data Capture, an electronic data capture tool, hosted by University of Iowa, only accessible to a designated research team member, to maintain unbiased physical examination and diagnostic testing.

### Statistical analyses: Brazil

Univariate analyses were performed to determine association between demographic variables and *Leishmania* spp. seropositivity. Pearson’s chi-square test or Fisher’s exact test were utilized to assess categorical variables. D’Agostino & Pearson normality test was performed to determine whether the age variable had a normal distribution. Upon identifying a non-normal distribution the Mann-Whitney test was used to assess age. Logistic regressions were performed to assess the association between tick-borne disease exposure and *Leishmania* spp. seropositivity controlling for sex, breed, physical appearance and tick serostatus. Older age, male sex, and breeds including Boxers, Italian Spinones, Corsicas, Foxhounds and Beagles have all been shown to have higher risk for *Leishmania* infection and disease progression [7, 30, 31]. Physical signs of leishmaniosis were included in the regression model to evaluate a correlation between clinical attributes and diagnostic positivity [23, 25]. As the Brazilian Ministry of Health recommends the use of two serological tests to confirm *Leishmania* spp. seropositivity we included an additional regression analysis utilizing these more stringent recommendations. Statistical analyzes were performed using SAS 9.4 (SAS Institute, Cary, NC, USA), Graph Pad Prism 6 (GraphPad Software Inc., La Jolla, CA, USA), and ArcGIS (Esri, Redlands, CA, USA). Statistical significance was defined as *P*-values at or below 0.05.

### Statistical analyses: USA

Demographic variables sex and age were compared against clinical leishmaniasis status (Leish+/Leish-) using Pearson’s chi-square test of independence. Previous research in our laboratory identified the average age at mortality as six years of age. Age was therefore categorized as dogs six years of age or younger or older than six. Since the observed and expected counts for age (≤ 6 years of age/> 6 years of age) were very small, we used Fisher’s exact test instead. Odds ratios were computed along with the corresponding 95% confidence intervals (CI). To further evaluate the association between serological positive tick-borne co-infections and the development of clinical leishmaniosis, a logistic regression was performed with clinical leishmaniosis status as outcome variable. All variables with a *P*-value of less than 0.1 from the univariate analyses were included in the logistic regression as predictor variables. In addition, sex and age were assessed as explanatory variables as both male sex and older age have been reported as potential risk factors related to CanL [7, 32]. Vaccine status was included as a potential explanatory variable as vaccination could reduce disease progression of CanL [25, 31]. Therefore, sex, age, region, vaccine status and number of serologically positive tick-borne diseases were used as explanatory variables with the outcome of interest as polysymptomatic CanL. Dogs that did not progress to polysymptomatic disease were considered controls.

Dogs that began the study as negative or asymptomatic for either tick-borne or CanL disease were included in this analysis. In the USA, the main route of transmission of CanL is vertical transmission. Due to this route of transmission, dogs are exposed to *Leishmania in utero*. While both qPCR and serological tests provide mostly accurate identification of dogs positive for infection/exposure *L. infantum*, there is still the potential for a false negative diagnostic test result. Dogs that began the study as negative and then became positive later may either have had a parasite load/immune response that was below detection during the first time-point or they may have been a true diagnostic false negative. If dogs diagnostically negative at enrollment but living in highly exposed groups were all excluded, the analysis would be biased to only include dogs that were potentially more ill. This would overestimate the true effect of tick exposure upon disease progression.

As this model utilizes longitudinal data, generalized estimating equations (GEE) were used to estimate regression model. An exchangeable correlation matrix structure and logit link function were utilized. Due to the low prevalence of CanL in this population, the rare disease assumption, which states that in cases of rare diseases the odds ratio and relative risk ratio are equivalent, was satisfied and odds ratios were reported as relative risk ratios [33]. Last observation carried forward was used for serological status if dogs died throughout the study and an endpoint leishmaniosis status identified due to mortality. Statistical analyzes were performed using SAS 9.4 (SAS Institute, Cary, NC, USA), Graph Pad Prism 6 (GraphPad Software Inc., La Jolla, CA, USA), and ArcGIS (Esri, Redlands, CA, USA). Statistical significance was defined as *P*-values at or below 0.05.

## Results

### Brazil study demographics

**Fig. 1:**
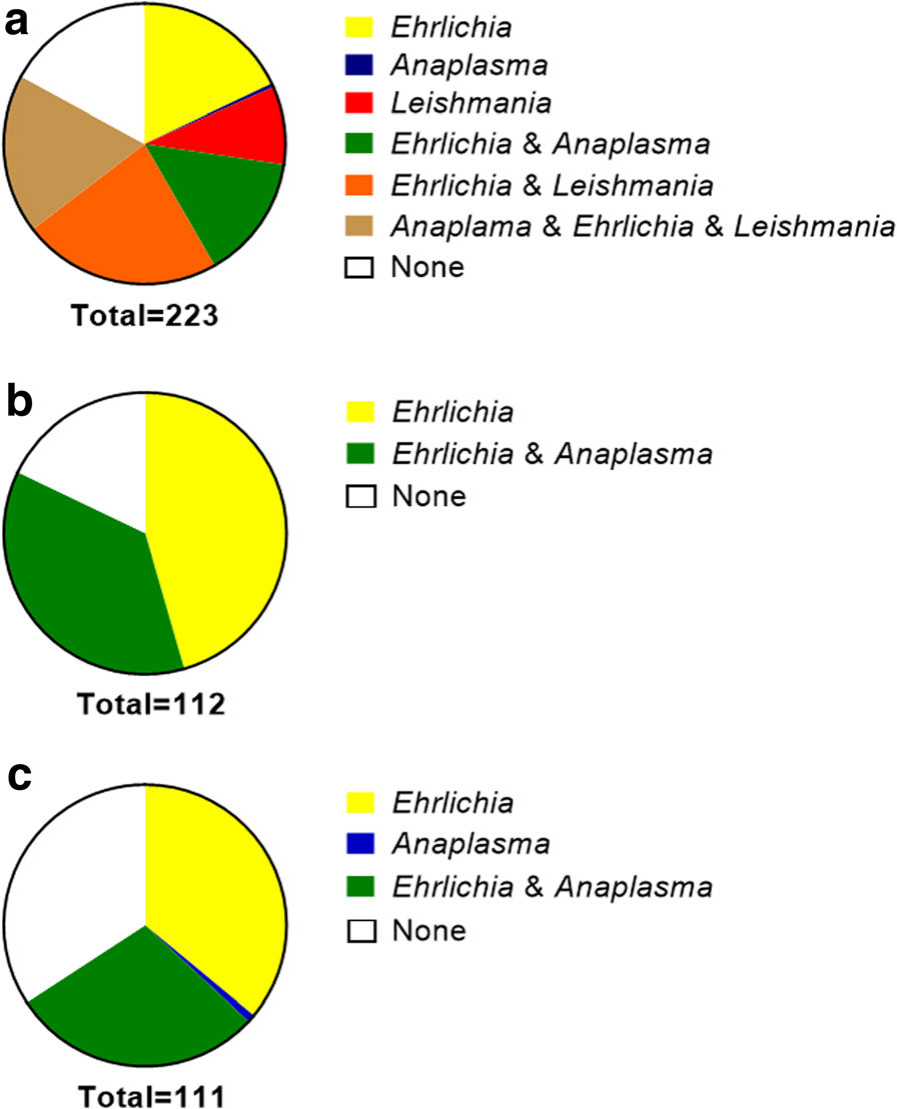
Distribution of *Leishmania* and tick-borne diseases in Brazilian dogs. **a** Distribution of tick-borne diseases and *Leishmania* seropositivity in full cross-sectional study cohort. Overall 82.96% of dogs were seropositive for one or more vector borne disease. **b** Tick-borne disease serostatus of dogs that tested positive *via* either ELISA and/or DPP® CVL for *Leishmania*. **c** Tick-borne disease serostatus of dogs that were negative *via* ELISA and DPP® CVL for *Leishmania* spp.

Understanding risk factors associated with disease progression and increased transmission are important for any public health control program aimed at reducing transmission from canine reservoirs to people. We performed a cross-sectional study of household dogs around Natal, Brazil, to determine the frequency and association of exposure to tick-borne diseases and the outcome of seropositive *L. infantum* infection. Blood/serum samples were collected from 223 dogs within a lower socioeconomic area of Natal, Brazil. Age and breed information was collected from dog owners. The average age was 2.5 years-old with a range from 3 months to 13 years (Table 1). Only a quarter of dogs were identified by veterinarians as showing visible signs of VL. The most common sign was onychogryphosis (17%), other signs were seen in less than 10% of dogs. Tick-borne disease exposure was high. *Ehrlichia* spp. was the most common tick-borne disease exposure. *Leishmania* spp. seropositivity was high with 50% of dogs testing seropositive on one or more tests (Fig. 1a).

**Table 1:** Demographics of Brazil cross-sectional study cohort

Variable	Value, *n* (%)
Sex, *n* (% male)	115 (51.57)
Age, mean ± SD (range) (years)	2.48 ± 2.16 (0.25–13.00)
Breed, *n* (% mixed breed)	188 (90.38)
Appearance	
Onychogryphosis	40 (17.94)
Cachexia	19 (8.52)
Wounds/skin lesions	18 (8.07)
Apathy	4 (1.79)
None	171 (76.68)
Tick serology	
*Ehrlichia* spp.	91 (40.81)
*Anaplasma* spp.	1 (0.45)
*Anaplasma* spp. + *Ehrlichia* spp.	73 (32.74)
None	58 (26.01)
*Leishmania* spp. serology	
Seropositive: three tests	
DPP®CVL, SLA, rk39	12 (5.38)
Seropositive: two tests	
DPP®CVL and SLA	17 (7.62)
DPP®CVL and rk39	19 (8.52)
Seropositive: one test	
DPP®CVL	108 (48.43)
SLA	19 (8.52)
rk39	22 (9.87)
Seronegative (all tests)	111 (49.78)

*Abbreviation*: *SD* standard deviation

To better understand the association between tick-borne disease exposure and *Leishmania* infection in Brazilian dogs, a univariate analysis assessing each demographic variable in relationship to the outcome of *Leishmania* spp. seropositivity was performed (Table 2). Our previous research has shown with a cohort of 130 dogs from the USA that when using ELISA as a gold standard, the sensitivity and specificity of the DPP®CVL is 73% and 80%, respectively [24]. Furthermore, several additional studies corroborate these findings and have shown that these type of diagnostic tests correlate [34–37]. Therefore, DPP®CVL assay results were used as a proxy for *Leishmania* spp. seropositivity in this analysis. The risk of positive *Leishmania* spp. serostatus based on tick-borne disease exposure was statistically significant. The risk of *Leishmania* spp. seropositivity was significantly greater among dogs with any tick exposure *vs* dogs with no tick exposures (RR: 1.55, *P* = 0.0135) (Fig. 1b, c).

**Table 2:** Univariate analysis of study cohort variables based on *Leishmania* serostatus. Pearson’s chi-square test and ANOVA were used to analyze categorical variables where appropriate

Variable	*Leishmania*+ (*n* = 108)	*Leishmania*- (*n* = 115)	*P*-value
Sex, *n* (% male)	54 (24.2)	61 (27.6)	0.6886
Age, mean ± SD (range) (years)	2.45 ± 1.81 (0.42–8.00)	2.50 ± 2.45 (0.25–13.0)	
Breed, *n* (% mixed breed)	11 (5.29)	9 (4.33)	0.6392
Appearance, *n* (%)			
Onychogryphosis	21 (19.4)	19 (16.5)	0.3691
Cachexia	8 (7.41)	11 (9.57)	
Physical wounds	9 (8.33)	9 (7.83)	
Apathy	0 (0)	4 (3.48)	
None	81 (75)	90 (78.3)	
Tick disease exposure, *n* (%)			
*Ehrlichia* alone	88 (81.5)	76 (66.1)	0.0290
*Anaplasma* alone	39 (36.1)	35 (30.4)	
*Anaplasma* + *Ehrlichia*	39 (36.1)	34 (29.6)	
None	20 (18.5)	38 (33.0)	

*Notes*: Age differences assessed *via* Mann-Whitney test. *Leishmania*+ are dogs that tested positive *via* the DPP®CVL assay. *Leishmania*- are dogs that tested negative *via* the DPP®CVL assay

*Abbreviation*: *SD* standard deviation

### Tick-borne disease exposure is significantly associated with *Leishmania* spp. exposure in a dose-dependent manner

We performed a logistic regression assessing the outcome of seropositivity for *Leishmania* spp. using the number of tick-borne disease exposures, age, sex, breed, appearance of onychogryphosis, cachexia, physical wounds, or apathy as covariates (Table 3). Only dogs with complete data for all explanatory variables were included in the analysis (included 208/223 dogs). Dogs exposed to *Ehrlichia* spp. and *Anaplasma* spp. were 2.69× more likely to also be seropositive for *Leishmania* spp. (Adjusted RR: 1.68, 95% CI: 1.09–2.61, *P* = 0.019) than dogs not exposed to tick-borne disease. Dogs seropositive for *Ehrlichia* spp. alone or *Anaplasma* spp. alone were also statistically significantly more likely to be seropositive for *Leishmania* spp. than non-tick-borne disease seropositive dogs (Adjusted RR: 1.60, 95% CI: 1.04–2.45, *P* = 0.032). Using the Brazilian Ministry of Health guidelines, i.e. reactivity on two serological tests as seropositive for *Leishmania* spp., dogs seropositive for one tick-borne disease were at a significantly higher risk of being *Leishmania* spp.-seropositive (Adjusted RR: 4.86, 95% CI: 1.16–20.3, *P* = 0.030) (Table 3). This significant association indicates that dogs from northeastern Brazil exposed any tick-borne disease are more likely to be seropositive for *Leishmania.*

**Table 3:** Dogs seropositive for a tick-borne disease are more likely to be seropositive for *Leishmania*. Parameter estimates were determined using logistic regression

Variable	ARR	95% CI	*P*-value^a^
(i) Leish serology 1 test			
Sex			
Male *vs* female	0.97	0.74–1.28	0.85
Age	0.97	0.91–1.04	0.38
Appearance			
Onychogryphosis	1.22	0.84–1.76	0.29
Cachexia	0.99	0.54–1.80	0.97
Physical wounds	1.11	0.71–1.74	0.63
Apathy	0.35	0.60–2.05	0.24
Tick disease serostatus			
positive for 1 *vs* 0	1.60	1.04–2.45	**0.032**
positive for 2 *vs* 0	1.68	1.09–2.61	**0.019**
positive for 1 *vs* 2	0.95	0.70–1.29	0.73
Breed			
Mixed *vs* Purebred	0.99	0.63–1.54	0.95
(ii) Leish serology 2 tests			
Sex			
Male *vs* female	0.56	0.26–1.20	0.14
Tick disease serostatus			
positive for 1 *vs* 0	4.86	1.16–20.3	**0.030**
positive for 2 *vs* 0	2.75	0.60–12.7	0.19
positive for 1 *vs* 2	1.76	0.77–4.05	0.18

^a^Bold indicates statistically significant variables

*Notes*: Leishmaniosis 1 test: *Leishmania*-positive are dogs that tested positive *via* DPP®CVL assay or ELISA. *Leishmania*-negative are dogs that tested negative *via* the DPP®CVL assay and ELISA. Predictor variables for this model included age, sex, appearance, tick disease status, and breed. Leishmaniosis 2 tests: *Leishmania*-positive are dogs that tested positive *via* the DPP®CVL assay and ELISA. *Leishmania*-negative are dogs that tested negative *via* the DPP®CVL assay and ELISA. Predictor variables for this model included sex and tick disease serostatus

*Abbreviations*: *CI* confidence interval, *ARR* adjusted relative risk

### Longitudinal study of tick-borne co-infection in *L. infantum*-infected dogs

Based on these findings in Brazilian dogs, indicating that there was substantial exposure to tick-borne diseases, and that this exposure increased the risk of seropositive VL, we were interested in learning whether this was a causal relationship. Specifically, we wanted to test in a setting where dogs were already infected with *L. infantum* how exposure to tick-borne diseases altered the risk of progression to CanL. As we have access to a large cohort of USA dogs infected *in utero* with *L. infantum* [4, 38] and highly exposed to tick-borne diseases, we used this group of dogs to follow longitudinal exposure to tick-borne disease and progression with VL. Two hundred eleven dogs were enrolled in a longitudinal nested case-control study stemming from a larger vaccine trial [25]. Dogs positive for a tick-borne infection *via* the 4DXSnap Plus test were on average 4.53 years-old with a standard deviation of 2.05 years, while dogs that were identified as tick disease-negative were slightly younger (3.80 years, SD 2.41 years, Table 4). No significant differences between age groups based on tick serology or age group based on sex were identified (Additional file 1: Figure S2). A univariate analysis of dogs that progressed to symptomatic disease, diagnostically positive with two or more clinical signs, *vs* those that did not, was performed. This analysis showed that dogs with historical exposure to two or more tick-borne disease co-infections *via* ELISA were 3.39 times more likely to have clinical signs for leishmaniosis (95% CI: 1.174–9.413, *P* = 0.0381). It was also seen that dogs older than six years of age were 3.62 times more likely to have clinical signs (95% CI: 1.427–8.715, *P =* 0.0055) (Table 5).

**Table 4:** Demographics of longitudinal study cohort. Combined IDEXX SNAP® 4DX® test results for antibody against *Ehrlichia ewingii* and *Ehrlichia canis*, *Anaplasma phagocytophilum* and *Anaplasma platys*, *Borrelia burgdorferi*, and antigen to *Dirofilaria immitis*

Variable	IDEXX SNAP® 4DX® + (exposed)	IDEXX SNAP® 4DX® - (unexposed)
No.	62	149
Age, % ≤ 6 years-old	79.03	88.51
Sex, % male	50.00	51.68

**Table 5:** Univariate analysis of factors leading to CanL from longitudinal study

Variable	Leish+ (*n* = 16)	Leish- (*n* = 195)	RR	*P*-value
Age, % ≤ 6 years old	62.50	87.69	3.62	0.0055
Sex, % male	43.75	51.79	0.7418	0.5360
Tick serology-historical exposure			3.388	0.0381
2 or more	41.67	15.87		
less than 2	58.33	84.13		
Treatment group	50.00	51.31	0.9519	>0.9999
Placebo *vs* vaccine	50.00	48.69		

*Abbreviations*: CanL, canine leishmaniosis. Leish+, showing 2 or more clinical signs of canine leishmaniasis; Leish-, showing less than two clinical signs of canine leishmaniasis or negative for *L. infantum via* qPCR or DPP®CVL assay; RR, unadjusted relative risk

### Tick-borne disease exposure was significantly associated with clinical leishmaniosis

To establish the effect of tick-borne disease on the outcome of CanL controlling for all other important variables, a logistic regression analysis was performed on the longitudinal data. Only dogs that began the study as negative or asymptomatic for CanL were included in this analysis to address progression; 203 dogs were included. Ehrlichiosis, Lyme disease, anaplasmosis and CanL have some overlapping clinical signs [38–40]. Due to this, only dogs identified as polysymptomatic for leishmaniosis, three or more clinical signs specific to leishmaniosis, were considered as having progressed to CanL.

Adjusting for other variables and using a more stringent definition for clinical CanL, dogs with three or more tick-borne diseases have 11 times greater odds of being polysymptomatic for leishmaniosis than dogs with no exposure to tick-borne diseases (Adjusted Relative Risk, ARR: 11.65, 95% CI: 1.22–110.99, *P* = 0.033) (Table 6). We found that this association was dose dependent; exposure to more tick-borne diseases increased the risk of progression to clinical CanL within the time of the study, an important feature in indicating a causal relationship. The odds of being polysymptomatic for CanL were 7.69 times greater for dogs exposed to three tick-borne diseases (or more) compared to those exposed to the group of dogs with only one tick-borne disease (ARR: 7.69, 95% CI: 1.39–43.48, *P* = 0.0200). Dogs exposed to three tick-borne diseases also saw a significant increase in the odds of being polysymptomatic with CanL compared to dogs with two tick-borne diseases (ARR: 8.33, 95% CI: 1.01–66.67, *P* = 0.0490).

**Table 6:** More exposure to multiple tick-borne diseases leads to worse CanL. Multiple logistic regression with outcome defined as polysymptomatic leishmaniosis; dogs diagnostically positive for *Leishmania infantum* with three or more specific signs for leishmaniosis

Variable	Adjusted relative risk	95% CI	*P*-value
Age			
≤ 6 years-old *vs* older	8.50	2.22–32.51	0.0018
Region			
Mid-west *vs* other	11.78	1.79–77.57	0.010
Serology (no. of tick-borne dz)			
1 *vs* 0	1.49	0.29–7.79	0.630
2 *vs* 0	1.44	0.25–8.40	0.690
3 *vs* 0	11.65	1.22–110.99	0.033
3 *vs* 1	7.69	1.39–43.48	0.020
3 *vs* 2	8.33	1.01–66.67	0.049
2 *vs* 1	1.04	0.27–3.96	0.950

*Notes*: Explanatory variables controlled for in the model were age, region, tick serological status (positive *vs* negative), sex and vaccine status. Only significant variables are shown

*Abbreviations*: CanL, canine leishmaniosis; CI, confidence interval; dz, disease

### Dogs with tick-borne diseases and *Leishmania* were significantly more likely to die

**Fig. 2:**
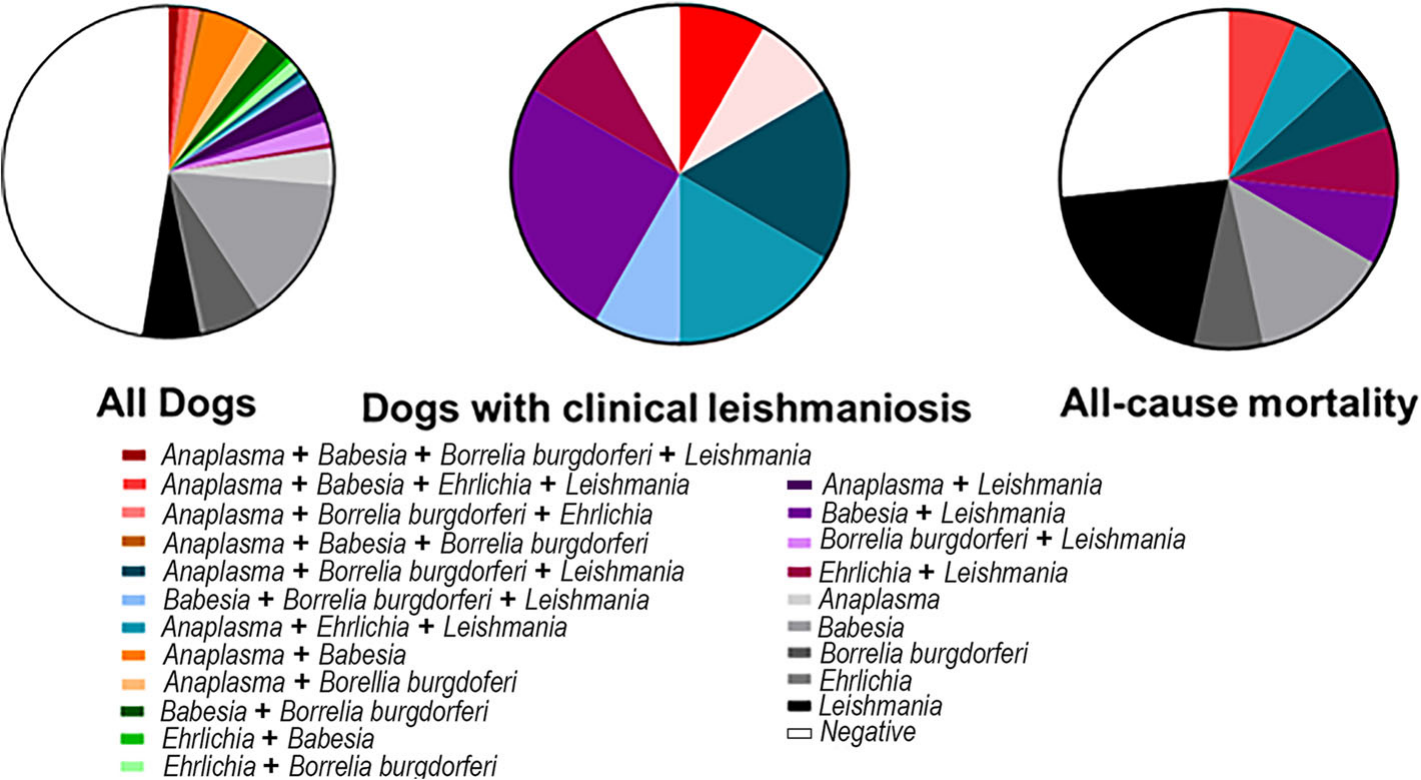
Distribution of tick-borne diseases based on CanL clinical status or mortality. Tick-borne disease exposure determined as positive or negative *via* ELISA. *Leishmania* spp. determined as diagnostically positive *via* qPCR and/or DPP® CVL. **a** Dogs with clinical leishmaniosis determined as dogs with three or more clinical signs of leishmaniosis and diagnostically positive for *Leishmania via* qPCR and/or DPP® CVL. **b** All-cause mortality determined as death for any reason

Antidotal evidence indicated that dogs with tick-borne disease diagnosis and a history of being diagnostically positive for CanL seemed to correlate strongly with a high mortality rate. Based on the proposed relationship between progression of clinical CanL after exposure to tick-borne diseases, we were interested to see how dogs diagnostically positive for *Leishmania* spp. and positive *via* ELISA for a tick-borne disease compared to those without these exposures in terms of all-cause mortality. Dogs diagnostically positive for *Leishmania* spp. and positive *via* ELISA for a tick-borne disease were 4.85 times more likely to die than those that negative for both (RR: 4.85, 95% CI: 1.65–14.24, *P* = 0.0051). When controlling for age, this relationship remained significant in dogs that were six years of age or younger (4.74 times more likely to die from all causes within one year (RR: 4.74, 95% CI: 1.32–16.88, *P* = 0.027)). This significant association between tick-borne disease exposure and CanL outcome or mortality can also be seen graphically (Fig. 2).

### *Ehrlichia* spp. and *Anaplasma* spp. cause most common tick-borne disease in dogs with clinical CanL

The most common tick-borne infection was *Babesia* spp. with an overall average seroprevalence of 31.65%. When evaluating dogs with clinical CanL (two or more clinical signs), *Ehrlichia* spp. and *Anaplasma* spp. were the most common tick-borne disease agent exposures among dogs with clinical CanL, of note also the two most common tick-borne disease agent exposures in Brazil as well. *Leishmania* spp. while only found in 17–20% of the study cohort depending on the time of the year was the most common exposure/infection found within dogs that died with more than 50% of the dogs that died being diagnostically positive for *Leishmania* spp. The second most common was *Babesia* spp. present in 27% of dogs that died (Table 7).

**Table 7:** Distribution of co-infections among dogs with clinical leishmaniosis and that died

Tick-borne exposure	% also CanL	% all-cause mortality
*Anaplasma* spp.	41.67	20.00
*Babesia* spp.	50.00	26.67
*Borrelia burgdorferi*	33.33	13.33
*Ehrlichia* spp.	41.67	20.00
*Leishmania* spp.	100	53.33

*Notes*: *Leishmania infantum* positive status was determined as any dog that was diagnostically positive *via* qPCR or is measured by either DPP® CVL assay or PCR-positive. Clinical leishmaniosis was determined as dogs with 2 or more clinical signs for leishmaniosis and a diagnostic positive test results on qPCR and/or DPP® CVL

### Other tick-borne infections

*Bartonella* spp. and *Rickettsia* exposure was found in the longitudinal study cohort as well. Three dogs were positive for *Bartonella* spp. infection *via* PCR at enrollment. All dogs were male, under the age of six, two of the dogs were from eastern USA, and one was from the Midwest. One dog of these dogs was also exposed to *Anaplasma* spp. and *Ehrlichia* spp.; another to *Anaplasma* spp., *Borrelia burgdorferi*, and *Babesia* spp., and one dog had been exposed to *Anaplasma* spp. and *Babesia* spp. The two dogs that tested positive for *Rickettsia* spp. *via* PCR tested positive in August were male and under the age of six from the Eastern USA. One dog was exposed to *Anaplasma* spp., *Borrelia burgdorferi* and *Leishmania* spp. The other dog was exposed to both *Borrelia burgdorferi* and *Leishmania* spp.

## Discussion

Co-infections can increase disease severity as is seen in both canine and human patients in Brazil with co-infections in addition to *Leishmania* infection [41, 42]. In Brazil, people infected with both *Leishmania* and human immunodeficiency virus (HIV) had increased mortality [43, 44], much like dogs in this study with tick coinfection and CanL. A similar trend was seen in human patients with Lyme disease and babesiosis co-infection [45–47]. Increased disease severity and pathogenicity was found in patients co-infected with *Leishmania* and intestinal helminths [48]. Malnutrition is also a risk factor of leishmaniasis stressing the importance of understanding these diseases within areas of lower socioeconomic status where malnutrition is common [49]. In canine patients, higher morbidity and mortality between vector borne diseases was observed in multiple case reports and experimental infection studies including co-infection with *Anaplasma* spp. and *Ehrlichia* spp. as well as *Babesia* spp. and *Leishmania* spp. [50, 51]. Recent studies in Europe have identified significant associations between CanL and other vector borne diseases [18, 20, 52]. These studies have been limited by greater complication of establishing temporality when there is vector borne transmission [7]. Most, if not all, dogs in the longitudinal USA study were vertically exposed to leishmaniosis, with limited exposure after gestation. This greatly improves the ability to know what disease came first [3, 4, 38]. Other routes of transmission have been suggested including sexual transmission or a possible role of ticks in transmission. As ticks are not biological vectors for *L. infantum*, as compared to sand flies, their only role could be in mechanical transmission and multiple incomplete feedings by a tick. There is no evidence that supports tick-borne transmission of *L. infantum*.

The aim of this study was to determine whether there is a significant, causal relationship between tick-borne disease exposure and the development of VL in dogs, the domestic reservoir for infection in Brazil. We found that having multiple tick-borne co-infections, which was common in Brazilian dogs, significantly increased the risk of CanL progression. This relationship was dose-dependent, i.e. the more tick-borne co-infections a dog had, the higher the risk of CanL progression and mortality. Ninety-two percent of dogs with clinical leishmaniosis were positive for a tick-borne disease and 58% of dogs with clinical leishmaniosis were positive for two or more tick-borne diseases. Thirty-three percent of dogs that died had two or more co-infections. These results provide epidemiological evidence that co-infection with a tick-borne disease may offset the delicate balance within the immune system during *Leishmania infantum* infection. Based on these findings, in many endemic/enzootic areas, times of great tick-borne disease prevalence could potentially drive the occurrence of more *L. infantum* parasitemic dogs and greater *L. infantum* transmission to people. Conversely, based on this causal relationship between tick-borne disease exposure and clinical CanL, controlling tick-borne disease may also control CanL.

*Ehrlichia* spp. and *Anaplasma* spp. were the most common tick-borne disease exposures within dogs that progressed to clinical disease found in over 40% of the dogs. This could be because *Ehrlichia* spp. can infect macrophages and can lead to remarkable proinflammatory then regulatory immunity disrupting the Th1 response needed for control of CanL [53–56]. Once inside phagocytes *Ehrlichia* spp. can downregulate IL-12, important for activation of CD4 T cells to release IFN-γ important in upregulating macrophage killing to remove *Ehrlichia* spp. and *Leishmania infantum* [57, 58]. *Ehrlichia* spp. can reduce autophagy allowing bacteria and *Leishmania* spp. to continue growing and replicating within the cell [59]. These co-infections have been shown to alter host immunity in ways that would allow *L. infantum* infection to thrive within phagocytes leading to clinical disease.

This study highlights the need to address reducing tick-borne disease exposure and co-infection rates in order to reduce clinical progression and its important correlate, transmission of *Leishmania infantum*. Xenodiagnosis studies addressing CanL transmission to the sand flies suggested that clinically apparent dogs were more likely to transmit *L. infantum* to the sand flies [60–62]. If tick-borne disease prevention and treatments can be utilized to reduce leishmaniosis disease progression, there may also be a correlation in reducing of *Leishmania infantum* transmission. These aspects need to be addressed for control and elimination of the disease in both human and animal populations.

This study was limited in the length of time of follow-up. Additional studies following dogs for a longer would allow evaluation of long-term effects of tick exposure over multiple seasons. Loss to follow up was also a limitation of the study as dogs were lost to follow-up for many reasons including drafting and retirement, which limited the ability to identify all dogs that became clinically ill. The loss to follow up likely lead to an underestimation of the true association, as dogs that were drafted were likely underperforming potentially due to illness.

## Conclusions

Significant associations between exposure to tick-borne infectious agents and progression of CanL were found in this study. These findings will require additional immunological studies to identify the specific immune mechanisms of how tick-borne diseases affect progression of CanL. Future studies could lead to targets for potential treatments and immunotherapies based on the loss of immunoregulation that may be associated with tick-borne coinfection during CanL. Based on the association of tick-borne disease with CanL progression, tick prevention and risk management could be realistic tools to reduce the seroprevalence of CanL within hunting dogs.

## Additional files

Additional file 1: **Figure S1.** US hunting dog longitudinal study timeline. Dogs were sampled three times designated by up and down arrows, over a tick season. Peak tick season for all kennel locations is designated by black. The bridge to tick season, dependent on seasonal variation and geographic location, shown in grey. **Figure S2.** Age and sex distribution of dogs based on tick-borne disease exposure at enrollment. Tick exposure based on SNAP® 4Dx® Plus Test. **a** Age distribution. **b** Sex distribution. *Abbreviations*: CanL: canine leishmaniosis; qPCR: quantitative polymerase chain reaction; RR: risk ratio; ARR: adjusted risk ratio; OR: odds ratio; CI: confidence interval. (ZIP 212 kb)
