# Supplementary figures and images for: Comorbid infections induce progression of visceral leishmaniasis
Source: Parasit Vectors. 2019 Jan 23;12:54. doi: 10.1186/s13071-019-3312-3 (PMC6345068; doi:10.1186/s13071-019-3312-3)

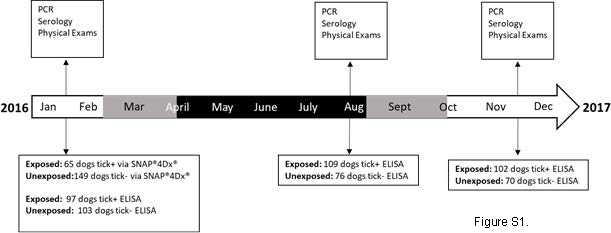

Supplement: Supplementary file 1 — Figure S1. US hunting dog longitudinal study timeline. Dogs were sampled three times designated by up and down arrows, over a tick season. Peak tick season for all kennel locations is designated by black. The bridge to tick season, dependent on seasonal variation and geographic location, shown in grey. Figure S2. Age and sex distribution of dogs based on tick-borne disease exposure at enrollment. Tick exposure based on SNAP® 4Dx® Plus Test. a Age distribution. b Sex distribution. Abbreviations: CanL: canine leishmaniosis; qPCR: quantitative polymerase chain reaction; RR: risk ratio; ARR: adjusted risk ratio; OR: odds ratio; CI: confidence interval. (ZIP 212 kb) [file 13071_2019_3312_MOESM1_ESM.zip › Figure S1.tif]

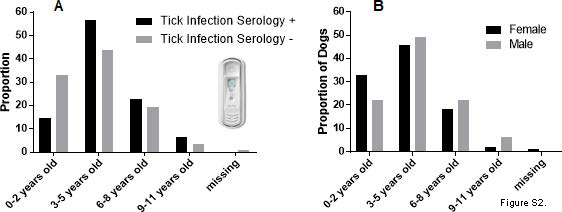

Supplement: Supplementary file 1 — Figure S1. US hunting dog longitudinal study timeline. Dogs were sampled three times designated by up and down arrows, over a tick season. Peak tick season for all kennel locations is designated by black. The bridge to tick season, dependent on seasonal variation and geographic location, shown in grey. Figure S2. Age and sex distribution of dogs based on tick-borne disease exposure at enrollment. Tick exposure based on SNAP® 4Dx® Plus Test. a Age distribution. b Sex distribution. Abbreviations: CanL: canine leishmaniosis; qPCR: quantitative polymerase chain reaction; RR: risk ratio; ARR: adjusted risk ratio; OR: odds ratio; CI: confidence interval. (ZIP 212 kb) [file 13071_2019_3312_MOESM1_ESM.zip › Figure S2.tif]
